# Biomarker Discovery and Molecular Docking Reveal *Marsdenia tenacissima* Fermentation Product’s Anti-Lung Cancer Components

**DOI:** 10.3390/cimb47060427

**Published:** 2025-06-06

**Authors:** Runtian Li, Lintao Li, Runzhi Li, Haiyang Wu, Guifang Dou

**Affiliations:** 1 Beijing Institute of Radiation Medicine, 27 Taiping Road, Beijing 100850, China; 2 School of Information, Renmin University of China, Beijing 100872, China

**Keywords:** *Marsdenia tenacissima*, UPLC-Q-TOF/MS, network pharmacology, TCGA, molecular docking

## Abstract

In traditional Chinese medicine, *Marsdenia tenacissima* is employed to prevent and treat lung cancer. The anti-tumor properties are further amplified by the fermentation product of *Ganoderma lucidum* and *Marsdenia tenacissima* (MGF). Nevertheless, the efficacy of the chemical components in combating lung cancer and the potential therapeutic targets for treating the disease remain ambiguous. UPLC-Q-TOF/MS was used to identify 19 components, all of which are unique C_21_ steroidal saponins found in MGF. The analysis of network pharmacology indicated that the active targets of these components were significantly concentrated in lung cancer and had a strong connection with cell proliferation. The bioinformatics analysis was conducted on data from TCGA and DisGeNET to identify a total of 28 biomarkers. Furthermore, our findings showed that the 19 targets connected to the active ingredients of *Marsdenia tenacissima* demonstrated significant enrichment in both the EGFR and apoptosis signaling pathways. Molecular docking technology was utilized to confirm the binding interactions of the primary constituents with the designated target.

## 1. Introduction

Lung cancer ranks as one of the prevalent forms of cancer worldwide, comprising 12.3% of all cancer cases [1]. The 2020 global cancer incidence statistics indicate that lung cancer accounts for 11.4% of all cancer diagnoses, placing it second in prevalence [2]. The challenging nature of treating lung cancer is attributed to its high rates of metastasis, recurrence, and complexity. Presently, numerous anti-lung cancer medications, including sorafenib and cisplatin, have been developed. Despite their contribution to enhancing lung cancer survival rates, these drugs are still plagued by issues such as limited efficacy duration, hepatotoxicity, nephrotoxicity, and the development of drug resistance [3]. Consequently, there is a pressing need for the exploration and creation of novel anti-lung cancer therapeutic agents that are characterized by enhanced safety profiles, heightened efficacy, reduced toxicity, and increased effectiveness.

The effectiveness of traditional Chinese medicine in treating lung cancer is well recognized among patients, largely because of its varied active ingredients, ability to target multiple anti-tumor pathways, and minimal occurrence of toxic side effects [4]. *Marsdenia tenacissima* (Roxb.) Moon [Apocynaceae; Marsdeniae tenacissmae caulis] is primarily found in the regions of Yunnan, Guangxi, Guizhou, and Sichuan in China. Additionally, it has been documented in other countries such as Vietnam, Laos, Myanmar, and Cambodia [5]. It predominantly thrives in montane forests situated at elevations ranging from 1500 to 2000 m [6]. Pharmacological research has demonstrated its efficacy in exhibiting anti-tumor, hepatoprotective, and anti-inflammatory properties [7,8]. *Marsdenia tenacissima* demonstrates promising anti-lung cancer properties and is commonly utilized in clinical settings. The primary active constituents responsible for its anti-tumor effects are C_21_ steroidal saponins; however, many of these components exhibit poor oral bioavailability and a limited absorption in humans [9]. Research suggests that C_21_ steroidal saponins, known for their potent anti-tumor effects, are primarily small polar molecules such as Tenacigenin A and Tenacigenin B. In the early stage of the research, the team utilized MGF, particularly through biotransformation, to generate a substantial amount of small polar aglycones [10,11]. Nonetheless, the exact mechanism by which these compounds combat lung cancer has not been thoroughly explored.

Scientists studying traditional Chinese medicine have achieved significant advancements in understanding the comprehensive effects of multi-channel, multi-target, and multi-component traditional Chinese medicine using network pharmacology [12,13,14]. This study systematically identified 14 C_21_ steroid saponin derivatives for the first time using UPLC-Q-TOF/MS technology, including 11 α-O-2-Methylbutyryl-12β-O-2-benzoyltenacigenin B and Tenacissimoside H. The structural characteristics of these complex compounds may be directly related to their enhanced anti-tumor activity. By integrating TCGA-LUAD (The Cancer Genome Atlas Program-lung adenocarcinoma) data, differential expression analysis, and multivariate Cox regression, a novel lung cancer prognostic risk model based on 11 genes was constructed. The AUC values for 1–3 year survival predictions all exceeded 0.7, demonstrating significantly better performance than traditional clinical indicators such as TNM staging. This study also established, for the first time, an association between the prognostic model of MGF and the tumor immune microenvironment. It was observed that the infiltration ratio of T helper cells and tumor-infiltrating lymphocytes was significantly increased in patients classified as low-risk, whereas the high-risk group predominantly exhibited macrophage infiltration. This suggests that an immunosuppressive microenvironment may drive the progression of lung cancer. This study provides a low-cost, high-precision prognostic assessment tool that can assist in clinical decision-making.

## 2. Materials and Methods

### 2.1. Preparation of MGF Methanol Extract and UPLC-Q-TOF/MS Analysis

MGF was prepared by Professor Tongxiang Liu’s research team at the School of Pharmacy, Minzu University of China (Beijing, China), by combining 0.2 g of the powder with 10 mL of methanol and leaving it for 30 min. The mixture was subsequently spun at 3000 rpm for 15 min, and the supernatant was collected. This step was repeated three times. The gathered extracts were subsequently mixed and dried with a nitrogen blower. The sample was dissolved in methanol to create a 2 mg/mL solution, which was subsequently filtered through a 0.22 μm filter prior to its utilization in UPLC-Q-TOF/MS analysis. The UPLC used ExionLC (AB Sciex, Concord, Ontario, Canada), the chromatographic analysis column was ACQUITY UPLC HSS T3 reverse column (2.1 × 100 nm 1.8 μm, Waters, Crop, Milford, MA, USA), and the mass spectrometer used SCIEX Triple TOF 5600+ to separate compounds at 25 °C with a flow rate of 0.2 mL/min. The solvents used were 0.1% formic acid in water (A) and acetonitrile (B), with a gradient from 100% to 0% A over 20 min. A 1 μL sample was injected, and the samplers were held at 4 °C. Using the ExionLC-MS with an ESI source, mass spectrometry was conducted, scanning 50–1200 *m*/*z*, with a sheath gas flow of 55 arb, a heater at 550 °C, spray voltages of ±4500 V, and collision energies of 20, 40, and 60 eV.

### 2.2. Data Collection

Based on the PubChem database and TCMSP database, MGF target genes were obtained. The chemical components of MGF from Table 1 were used to predict drug properties and oral absorbability using Swiss Target Prediction (http://www.swisstargetprediction.ch/, accessed on 25 February 2025) and then the disease targets associated with the drug components were compiled. Disease target genes of lung cancer were collected from databases such as the Gene Cards database (https://www.genecards.org/, accessed on 25 February 2025), Online Mendelian Inheritance in Man (OMIM) (https://omim.org/, accessed on 25 February 2025), and BATMAN-TCM (http://bionet.ncpsb.org.cn/batman-tcm/, accessed on 25 February 2025). The intersection genes were identified by determining the common genes between drug targets and disease targets. The training gene set was obtained from TCGA-GDC (https://portal.gdc.cancer.gov/, accessed on 25 February 2025).

### 2.3. Core Gene Analysis

The TCGA-GDC database was accessed to obtain clinical details, including stage, gender, and age, as well as gene expression profiles for individuals diagnosed with LUAD (https://portal.gdc.cancer.gov/, accessed on 25 February 2025). The ‘transcriptome profiling’ dataset was selected from the Files directory, the ‘RNA-Seq’ experimental method was chosen and added to the cart, and the data were downloaded. There are 633 instances in the cancer tumor category and 63 in the normal category. The “limma” package was used to perform differential expression analysis on the intersection genes and lung cancer-related RNA, with *p* < 0.05 and |log2(foldchange)| > 1 as the criteria for differential expression. The ‘Cluster Profiler’ was used to conduct KEGG and GO enrichment analyses on related genes. To identify genes associated with patient outcomes, univariate and multivariate Cox regression analyses were conducted using gene expression data from fermented drugs and clinical data from patients. Based on the genes screened by multivariate Cox regression analysis, a molecular prognostic model for lung cancer based on fermentation products was constructed. The risk prognostic equation was established according to the formula reported in the literature, that is, risk score = β1 × mRNA1EXP + β2 × mRNA2EXP + … βn × mRNAnEXP. β is the multivariate regression coefficient of the corresponding mRNA, and mRNAnEXP is the expression level of the corresponding mRNA. Correlation testing on genes related to patient prognosis was conducted.

### 2.4. Construction and Screening of Drug–Target Interaction Network

The intersection of drug component and disease target genes was then input into the STRING database. (https://string-db.org/, accessed on 25 February 2025) and the Protein–Protein Interaction (PPI) network was obtained. The PPI network was imported into Cytoscape 3.9.1 to construct a visual network diagram. With the Network Analyzr tool, topological analysis was performed, using degree, betweenness centrality, average shortest path, and closeness centrality as reference points. By arranging the degree values, genes scoring higher than the average are chosen as key wild points. An interaction network was built with these key targets as nodes, followed by a topological analysis of the top 10 targets.

### 2.5. Functional Enrichment Analysis of the Drug–Target Network

The R package ‘cluster Profiler’ was used to perform KEGG and GO enrichment analyses to explore the essential role and regulatory mechanisms of core genes in cellular function. The pathways exhibited significant enrichment when applying a statistical threshold of *p* < 0.05 and FDR < 0.25.

### 2.6. Molecular Docking Studies

**Figure 1:**
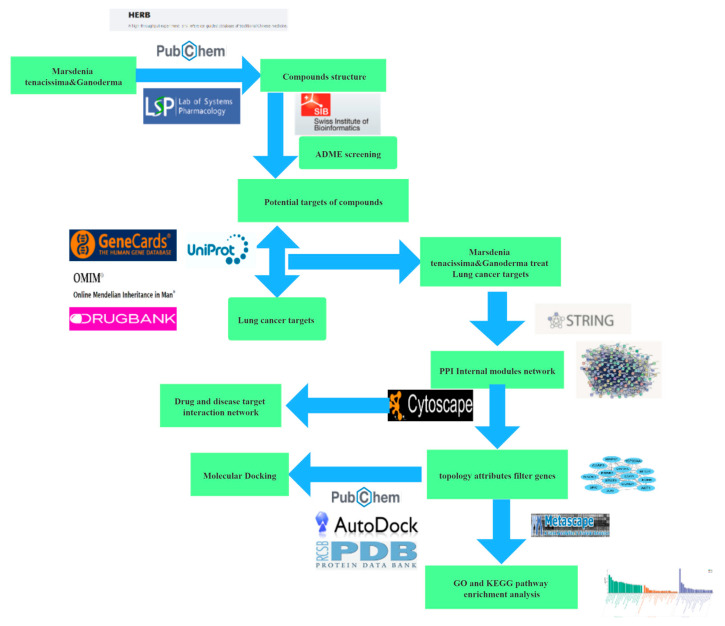
Network pharmacology process.

The molecular docking analysis was carried out using AutoDock 1.5.6, and the crystal structures of PI3K, mTOR, Rap, Ras, Casp3, Casp9, EGFR, VEGF, and MAPK1 were sourced from the RCSB Protein Data Bank. Before docking, polar hydrogen atoms were incorporated, and solvation parameters were allocated. Ligand substructures were isolated, and water molecules were taken out before the procedure. Molecules are more stable when the docking binding energy is lower. The binding poses and interactions of four drug candidates with four proteins were obtained with Autodock Vina v.1.2.2 and binding energy for each interaction was generated. The workflow of network pharmacology and molecular docking analysis is shown in Figure 1.

### 2.7. MD Simulation

MD simulations were conducted using GROMACS 5.1.5. Using the ACPYPE script, the ligand topology was created with the AMBER force field, and the AMBER99SB-ILDN force field was applied to the protein. A triclinic lattice containing TIP3 water molecules was used, and NaCl ions were added to neutralize the system. The complex was subjected to 1000 minimization steps followed by 100 ps of NVT and NPT equilibration. For 100 nanoseconds, simulations were performed at 310 K and 1.0 bar, employing periodic boundary conditions. The simulation box maintained a minimum distance of 1.0 nm from the protein, with a time step of 2 fs. Energy minimization utilized the steepest descent method, applying a 1.4 nm cutoff for Coulomb and van der Waals forces.

## 3. Results

### 3.1. MS Data Processing

As shown in Table 1, a total of 14 C_21_ steroid saponins were identified from the MGF, and their composition was compared with a database using MS exact mass, MS/MS fragmentation, and literature criteria. All identified components exhibit a quality precision of less than 5 ppm when compared to their theoretical values.

### 3.2. Identification and Analysis of DEGs

**Figure 2:**
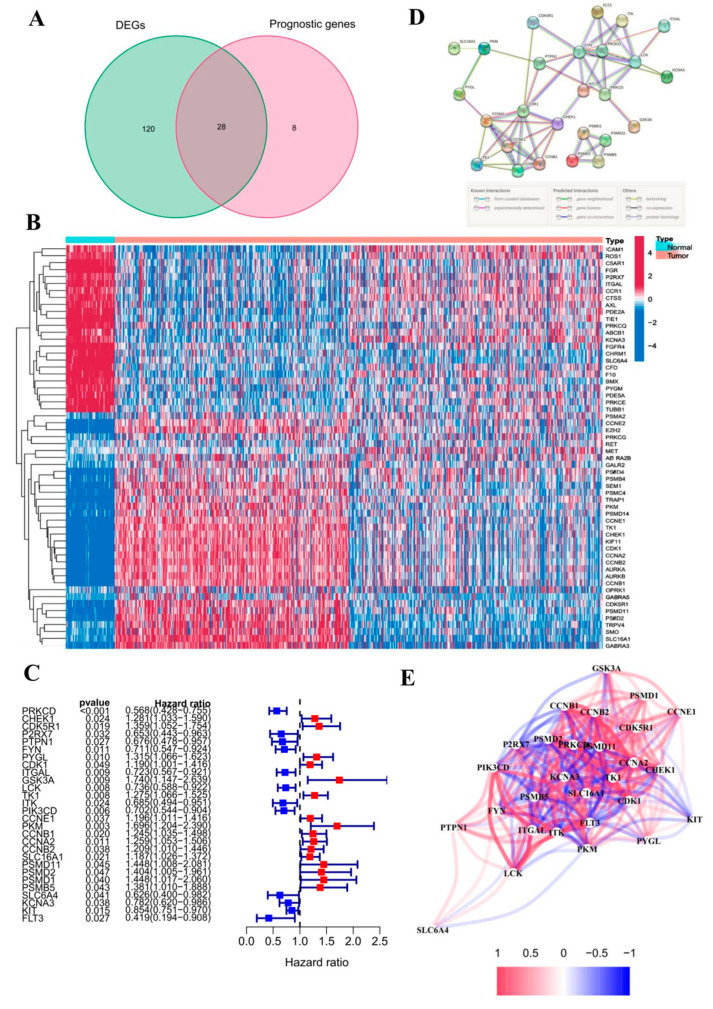
(**A**): intersection of differentially expressed genes and prognostic genes; (**B**): the prognostic differential genes were clustered in the tumor samples to analyze the heat map; (**C**): prognostic differential gene forest map (**D**): PPI network from STING database. (**E**): correlation network of core genes.

Through the investigation of genes involved in drug targets and diseases, a total of 216 genes were identified, with 209 genes found to be associated with cancer prognosis in TCGA-LUAD. Furthermore, a differential expression analysis was conducted on 633 samples of LUAD and 63 non-tumor tissues within the TCGA cohort. Using the R 4.5.0 software package “limma”, 148 differentially expressed drug–disease intersection genes (DEGs) were found in 63 normal tissues and 633 LUAD tissues, and 36 prognostic genes were obtained after intersection with prognostic genes. By intersecting the sets of DEGs and genes related to prognosis, we were able to identify potential biomarkers for disease prognosis. A VEEN diagram was drawn (Figure 2A) to obtain 28 prognostics differentially expressed genes (16 genes upregulated and 12 genes downregulated); see forest plot 1-C. By using 28 prognostic differential genes combined with TCGA-LUAD samples to draw, a sample and gene clustering heat map (Figure 2B) can be generated to identify genes exhibiting differential expression between normal and tumor groups. The heat map shows that genes such as CHEK1, PIK3CD, and PYGL are upregulated in tumor tissues. Flowing the import prognostic differential genes into STRING, the PPI topological network was predicted. The nodes represent genes or proteins, and connections between nodes indicate interactions between proteins (Figure 2D). The correlation network can be obtained through expression data, and the correlation coefficients between genes can be mined using text mining methods to construct a correlation network (Figure 2E).

### 3.3. Analysis of the GO and KEGG

**Figure 3:**
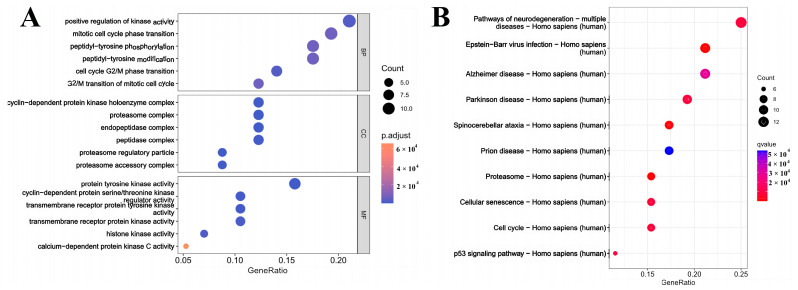
(**A**): GO enrichment analysis; (**B**): KEGG pathway.

To explore the MGF genes in LUAD, GO and KEEG pathway analyses were conducted on the 28 genes that were previously examined. The analysis of the biological process (BP) highlighted that this gene is vital in positively influencing kinase activity, assisting mitotic cell cycle phase transitions, and enhancing peptidyl-tyrosine phosphorylation (Figure 3A). Examination of Cellular Component (CC) analysis indicated a close association with the cyclin-dependent protein kinase holoenzyme complex, proteasome, and endopeptidase. Molecular function (MF) analysis demonstrated a strong correlation with protein tyrosine kinase activity, cyclin-dependent protein serine/threonine kinase regulator activity, and transmembrane receptor protein tyrosine kinase activity. The findings from the KEGG signaling pathway analysis indicate that the identified genes have the potential to influence various pathways, including those related to neurodegeneration, proteasome function, cell senescence, and cell cycle regulation (Figure 3B).

### 3.4. Prognostic Model Validation Analysis

**Figure 4:**
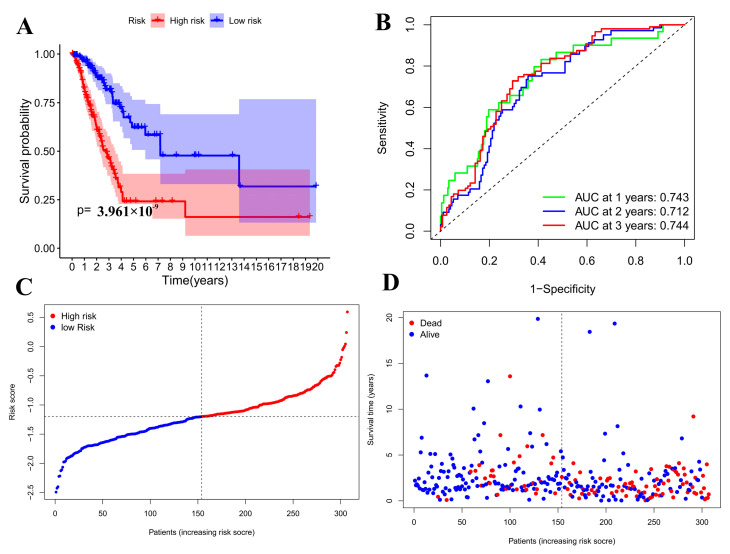
Model prediction. (**A**): survival curve; (**B**): ROC curve (**C**): the risk curve is red for high risk and blue for low-risk; (**D**): the risk curve is red for dead patients and blue for survival patients.

Based on each patient’s calculated median risk values, they were sorted into high-risk and low-risk groups, and a survival curve was developed (Figure 4A). With the passage of time, the survival rate of patients drops, indicating a significant divergence in outcomes between high-risk and low-risk groups. The model that was developed afterward allows for predicting how long a patient will survive. To estimate the precision of the patient survival model, a Receiver Operating Characteristic (ROC) curve was plotted. The ROC curve plots the false positive rate, and the vertical axis is the true positive rate; the AUC for predicting the one-, two-, and three-year survival rates of lung cancer patients were determined to be 0.743, 0.712, and 0.744, respectively (Figure 4B), indicating that the model has high accuracy, and that the sensitivity and specificity of the model gradually increase over time.

A multivariate Cox analysis was employed to determine the regression coefficients of 11 genes, leading to the development of a risk model. The risk calculation formula was derived from this analysis: risk value= (−0.34151) *PRKCD expression + … + (−0.08685) *KIT expression. Figure 4C shows the relationship between multiple factors and prognosis. The *p*-values of staging and risk coefficient are both less than 0.05, indicating that the two factors do not interfere with each other and that the established model meets the requirements. Patients can be categorized into low-risk and high-risk groups based on their risk score and survival status, using the median risk value as a dividing line, and risk curves 4-C and 4-D are plotted. As depicted in Figure 4C, the risk value rises from left to right, and Figure 4D reveals that the number of deaths also rises in that direction. Thus, the model suggests that an increase in risk leads to an increase in deaths.

**Figure 5:**
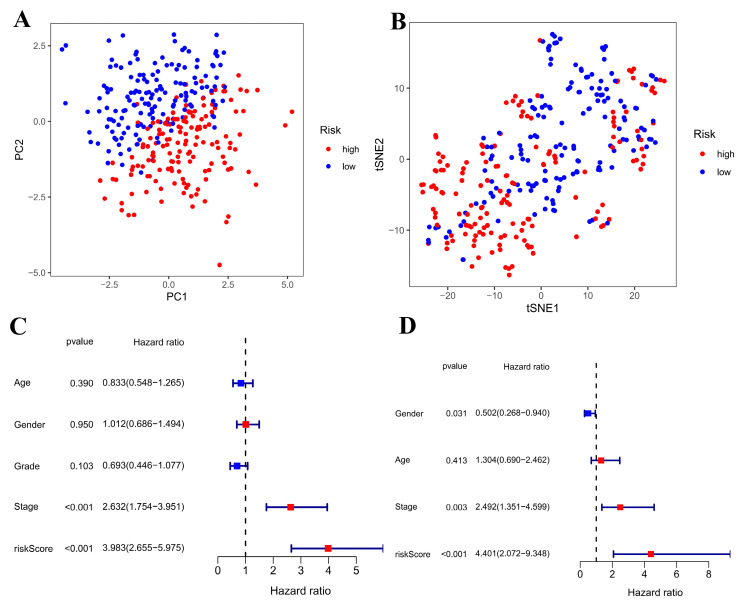
Model prediction. (**A**): PCA analysis chart; (**B**): T-SNE analysis; (**C**): single factor Cox analysis forest map (**D**): multifactor Cox analysis forest map.

The model’s 11 gene expression levels were visualized and plotted using PCA and t-SNE analysis (Figure 5A,B). It is evident that patients are categorized into high-risk and low-risk groups, showing that the predictive model can fully distinguish between them.

Using the transcriptome and survival data from TCGA-LUAD, a univariate Cox analysis was conducted on 28 genes with varying prognostic outcomes, identifying 11 genes linked to patient survival status and duration. Prognostic analysis was performed based on patient age, gender, disease stage, and disease grade (Figure 5C). It can be seen from Figure 5C that *p* < 0.05 for disease stage (Stage), and the disease risk level (risk score) indicates that these two factors have a significant relationship with disease prognosis. It can also be seen that the Hazard radio (HR) of age is greater than 1, indicating that age is positively correlated with disease prognosis, and the older the age, the greater the risk of disease prognosis; the HR of gender is less than 1, indicating that the risk of males is greater than that of females; it can also be seen from Figure 5D that as the stage of the disease increases, the risk of the disease increases, which also means that the risk of lung cancer patients in stages III and IV is greater than that of patients in stages I and II; the prognostic risk of patients with risk coefficients in III and IV is significantly greater than that of patients in stages I and II.

### 3.5. Analysis and Evaluation of Tumor Immune Cell Infiltration and Immune Environment

**Figure 6:**
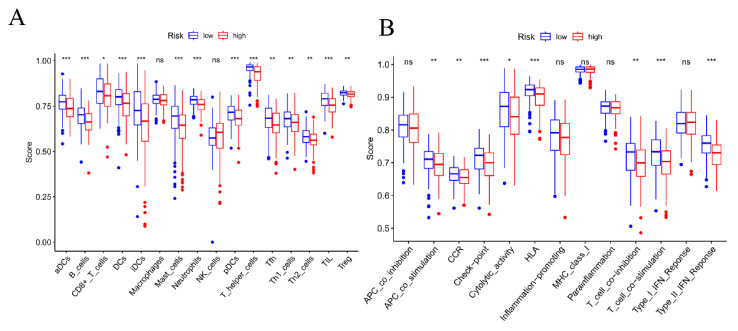
(**A**): Immunocyte correlation (**B**): immunofunction correlation. * *p* < 0.05, ** *p* < 0.01, *** *p* < 0.001.

An immune score correlation analysis was carried out to investigate the occurrence and development of immune cell infiltration in lung cancer, aiming to clarify the proportion of each immune cell in the tumor and the function of immune responses. Figure 6A shows that aDCs cells, T-helper cells, TIL cells, and iDCs cells account for the highest proportion in tumors, and all have the largest proportion in low-risk areas; their main functions are also in T cell stimulation and immune response.

### 3.6. Network Pharmacology Analysis with “Ingredient-Target-Disease”

**Figure 7:**
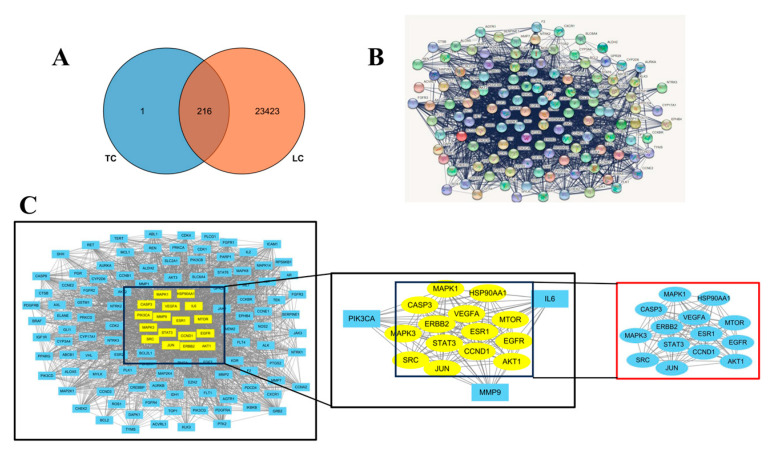
Network pharmacology result (**A**): drug target and disease target Ven diagram; (**B**): PPI topology network; (**C**): core target screening.

The analysis of fermentation products from MGF identified 217 targets in total. Following the removal of duplicate lung cancer targets sourced from Genecards, OMIM, and DrugBank databases, a total of 23,540 genes were compiled. Subsequently, through the comparison of compound targets and disease targets, 216 common targets were identified as potential targets for the treatment of lung cancer using the MGF, as illustrated in Figure 7A.

After the intersection targets were loaded into the STRING database and the human species was chosen, an automatic generation of a protein–protein interaction network occurred. Target genes that were not connected to the network were subsequently removed, resulting in the creation of Figure 7B. The STRING data were then imported into Cystoscope for further analysis of the network, leading to the identification of core targets (Figure 7C): SRC, JUN, AKT1, EGFR, MTOR, HSP90, CASP3, MAPK1, VEGFA, STAT3, ERBB2, and ESR1.

**Figure 8:**
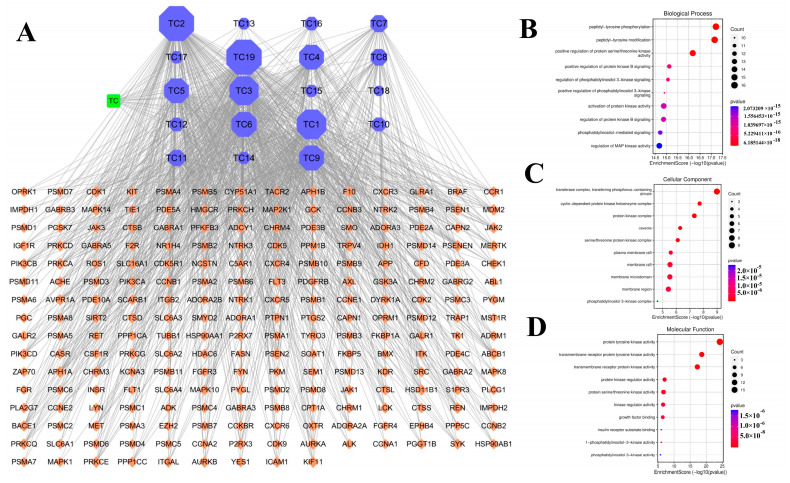
GO analysis bubble diagram (**A**): “drug component-target-disease” network; blue is the drug component, orange is the common target of drug and disease, and green is the drug. (**B**): biological process BP; (**C**): cell process CC; (**D**): molecular function MF.

Upon importing the data into Cystoscope, a network diagram was generated to visualize the “Ingredient-Target-Disease” relationships. (Figure 8A).

### 3.7. GO Enrichment Analysis and KEGG Pathway Analysis

A total of 215 GO entries were uncovered through the GO biological enrichment analysis, which included 162 entries related to biological processes BP, 26 to CC, and 27 to MF. Specifically, the biological processes were mainly linked to peptidyl tyrosine phosphorylation, modification of peptidyl tyrosine, enhancement of protein serine/threonine kinase activity, and the positive regulation of protein kinase B signaling, among others. The regulation of the cell cycle primarily involves the movement of phosphorus-containing groups by the transferase complex, the cyclin-dependent protein kinase holoenzyme complex, the protein kinase complex, and the serine/threonine protein kinase complex. Meanwhile, molecular function mainly includes activities like protein tyrosine kinase activity, transmembrane receptor protein tyrosine kinase activity, transmembrane receptor protein kinase activity, and protein kinase regulatory activity.

**Figure 9:**
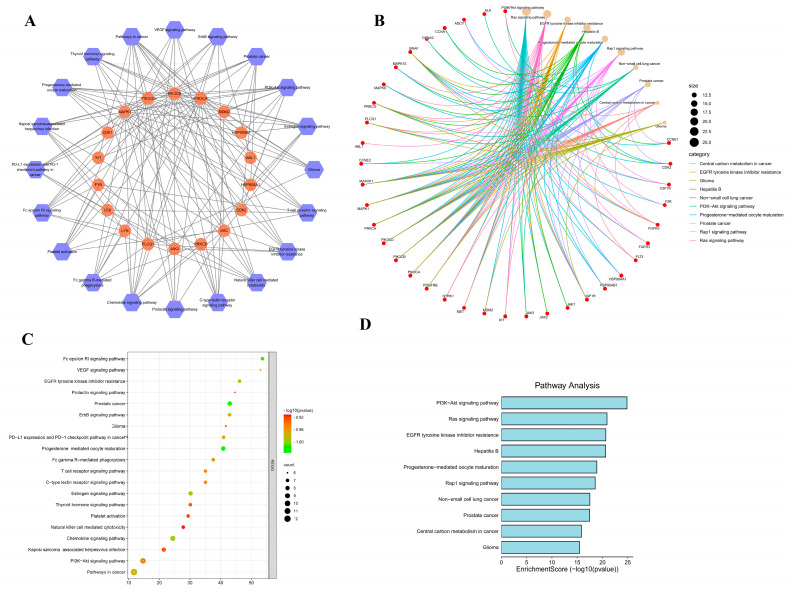
KEGG enrichment analysis (**A**): target–path interaction network (**B**): target–path correlation coefficient network (**C**): KEGG bubble chart; (**D**): KEGG histogram.

A total of 20 pathways were found to be enriched in the KEGG pathways (Figure 9). Among them was EGFR tyrosine kinase inhibitor resistance [15], PD-L1 expression, and PD-1 checkpoint pathway in cancer [16]; the T cell receptor signaling pathway and PI3K-Akt signaling pathway are closely associated with lung cancer and are therefore considered the main pathways of interest for further research [17,18].

### 3.8. Molecular Docking Simulation Verification

**Figure 10:**
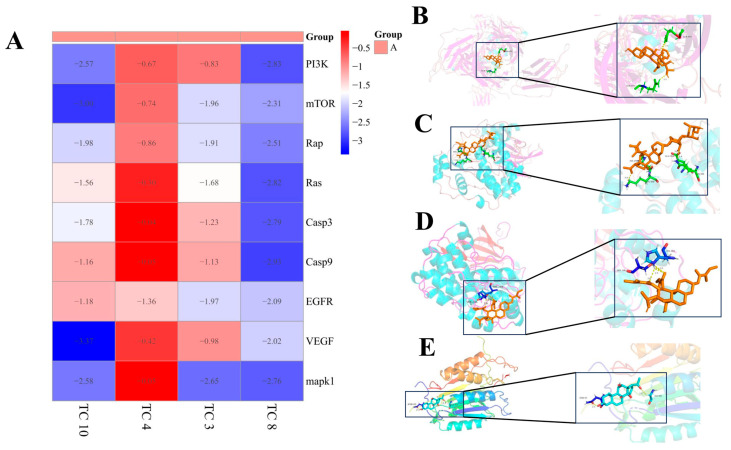
Docking mode of receptor proteins and active ingredients: (**A**): the heatmap illustrating the magnitude of binding energy of components in TP with respect to core target proteins. (**B**): Tenacigenin A-VEGF; (**C**): 11α-O-Tigloyl-12β-O-benzoyl-marsdenin-EGFR; (**D**): 11α, 12β-Di-O-tigloyltenacigeninB-MAPK1; (**E**): 11α-O-Tigloyl-17β-tenacigenin B-Casp9.

According to the KEGG analysis results and the core target protein screening analysis, the cancer cell proliferation pathway and cancer cell apoptosis pathway-related proteins of the classical pathway were selected, including PI3K, mTOR, Rap, Ras, Casp3, Casp9, EGFR, VEGF, and MAPK1 as target proteins to be docked [19,20,21]. The proteins of interest were obtained from the PDB database, and Autodock_vina software was used to compute the minimum binding energy between these nine proteins and the predicted active ingredients. To assess the affinity of the components of MGF for the core target, we performed molecular docking analyses. We calculated the binding energies of three components interacting with four proteins using Autodock Vina v.1.2.2. The results are shown in Figure 10A. The docking outcomes, presented in Figure 10B–E, indicate that the active ingredients can create hydrogen bonds and van der Waals forces with the ligand protein.

### 3.9. Molecular Dynamics Simulation of Protein–Ligand Complexes

**Figure 11:**
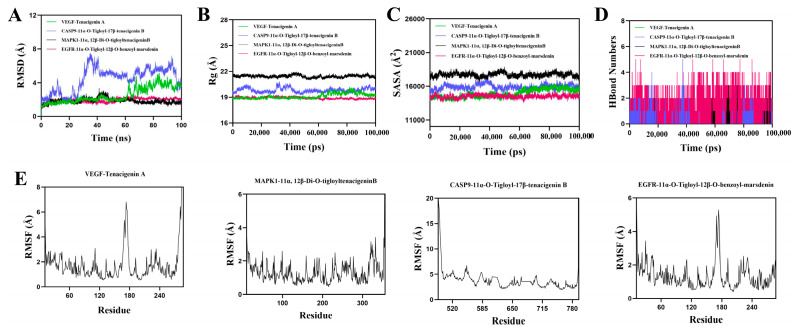
Molecular dynamics simulation of protein–ligand complexes. (**A**): RMSD values of protein–ligand complexes over time; (**B**): Rg values of protein–ligand complexes over time; (**C**): SASA values of protein–ligand complexes over time; (**D**) HBonds values of protein–ligand complexes over time; (**E**): RMSF values of amino acid skeleton atoms in protein–ligand complexes.

RMSD is an effective measure of protein and ligand conformational stability, with lower values indicating better stability. The simulation system’s balance was assessed using RMSD. Figure 10A shows that, except for the CASP9-11 α–O-Tigloyl-17 β–tenacegenin B complex, the VEGF Tenacigenin A complex system remained stable between 5 ns and 60 ns, with a slight increase after 60 ns, staying below 3.4 Å overall. The MAPK1-11 α, 12 β–Di-O-TigloyltenacigenB complex stabilized after 50 ns, fluctuating around 1.5 Å, while the EGFR-11 α–O-Tigloyl-12 β–O-benzoyl-masdenin complex stabilized after 10 ns, fluctuating around 1.9 Å. The low RMSD values for MAPK1-11 α, 12 β–Di-O-TigloyltenacigenB and EGFR-11 α–O-Tigloyl-12 β–O-benzoyl-masdenin indicate high stability when bound to MAPK1 and EGFR proteins. The radius of gyration (Rg) indicates changes in protein structure and tightness; larger Rg changes suggest a more swollen system. Analysis showed that the VEGF-Tenacigenin A, MAPK1-11 α, 12 β-Di-O-TigloylTenacigenin B, and EGFR-11 α-O-Tigloyl-12 β-O-benzoyl-mrsdenin complex exhibited fluctuations before stabilizing. This small molecule–protein complex undergoes conformational changes during movement (Figure 10B). The solvent accessible surface area (SASA) measures protein surface area. In this simulation, SASA between the target protein and small molecules was calculated (Figure 10C). Results indicated that the VEGF-Tenacigenin A, MAPK1-11 α, 12 β-Di-O-TigloylTenacigeninB, and EGFR-11 α-O-Tigloyl-12 β-O-benzoyl-masdenin complexes showed slight fluctuations, demonstrating that small molecule binding can alter the binding microenvironment and change SASA. Hydrogen bonds are crucial for ligand–protein binding. Figure 10D shows the number of hydrogen bonds formed between small molecules and target proteins. VEGF Tenacigenin A and MAPK1-11 α, 12 β–Di-O-TigloyltenacigenB typically form around one hydrogen bond, ranging from 0 to 3. EGFR-11 α–O-Tigloyl-12 β–O-benzoyl-mrsdenin usually forms about four hydrogen bonds, ranging from 0 to 5. This suggests strong hydrogen bonding interactions between these ligands and their target proteins. RMSF indicates the flexibility of protein amino acid residues. Figure 11E shows that the RMSF values for the VEGF-Tenacigenin A, MAPK1-11 α, 12 β–Di-O-TigloyltenacitinB, and EGFR-11 α–O-Tigloyl-12 β–O-benzoyl-masdenin complexes are generally low (mostly under 0.3 Å), suggesting reduced flexibility and increased stability.

## 4. Discussion

LUAD is an aggressive neoplasm that arises from the lungs. Due to its elusive nature, the majority of cases are diagnosed in advanced stages, posing challenges for effective therapeutic interventions. *Marsdenia tenacissima*, a monotherapeutic agent with wide-ranging anti-cancer properties and minimal toxicity, has the potential for commercial distribution in the pharmaceutical market [22]. The enhancement of anti-tumor activity in *Marsdenia tenacissima* following fermentation with *Ganoderma lucidum* underscores the importance of investigating its molecular mechanism of action [10].

In the bioinformatics study, a clinical prognostic risk model of 11 target genes of MGF was constructed, and its relationship with peripheral immunity was explored, and potential target indicators were further confirmed. First, 216 genes were collected by taking the intersection of drugs and diseases, and then 28 genes were obtained through difference and prognosis analysis. The analysis of these 28 genes revealed their association with the cell cycle, cellular aging, and the activation and release of certain enzymes. Through additional univariate and multivariate Cox analysis, 11 models for prognostic risk were acquired. The expression analysis of 28 genes demonstrated their role in significant biological processes, including the cell cycle, cellular senescence, and the regulation of enzyme activation and release. The identification of 11 prognostic risk models was achieved through subsequent univariate and multivariate Cox analyses, which classified clinical patients into high-risk and low-risk categories. It has shown that CDK5R1 may be related to prognosis [20]. PTPN1 is intimately linked to the cell cycle and is also associated with cell migration and movement [23]. Research has shown that the FYN gene is significantly expressed in tumor samples but has low expression in normal tissues, making PYN a potential new target for anti-lung cancer studies. PI3CD, a subtype of PI3K, plays a significant role in cancer cell proliferation and growth. Inhibiting its expression can effectively reduce cancer cell proliferation [24].

Studies on immune infiltrating cells in LUAD showed that macrophages had a higher infiltration ratio in the high-risk group compared to the low-risk group, while T-helper cells had a lower ratio. In the examination of prognostic models, a significant number of functions related to immune evasion are implicated, with the human leukocyte antigen (HLA) playing a crucial role. A mechanism by which tumors bypass the host’s immune surveillance is the loss of HLA class I antigen haplotypes, a phenomenon observed in various tumor cell types. Research has indicated that cytotoxic T lymphocytes are capable of eliminating tumor cells displaying normal HLA class I expression during the initial phases of carcinogenesis, while only tumor cells exhibiting haplotype loss of HLA class I antigen are able to evade immune surveillance and progress into clinically detectable cancer [25]. In conclusion, the 11 fermentations’ drug-related genes identified through bioinformatics techniques demonstrate significant predictive utility for the prognosis of LUAD, potentially serving as a foundation for personalized prognostic assessment and immunotherapeutic strategies.

After using the Swisstarget database to predict drug availability, the active components of *Marsdenia tenacissima* saponins were identified, including 11α-O-2-Methylbutyryl-12β-O-2-benzoyltenacigeninB, 11α-O-2-Methylbutyryl-12β-O-2-tigloyltenacigeninB, 11α, 12β-Di-O-tigloyltenacigeninB, 11α-O-Tigloyl-12β-O-benzoyl-marsdenin,11α, 12β-Di-O-tigloyltenacigeninB, and 11α-O-Tigloyl-12β-O-benzoyl-marsdenin. These aglycones show strong anti-cancer properties. The targets screened out through the “Ingredient-target-disease” target are mainly related to cancer cell proliferation, cell apoptosis, and cell membrane epidermal receptor factors. Research on the PI3K target is imperative due to its ability to regulate the proliferation and growth of cancer cells. Research indicates that *Marsdenia tenacissima* extract reduces melanoma cell viability and triggers apoptosis by modulating the PI3K/AKT/mTOR signaling pathway [26]. HSP90 is more abundant in cancer cells than in normal cells, and inhibiting it can slow cancer cell growth and trigger cell death [27]. The SRC gene is associated with cancer cell migration and invasion. Research indicates that salidroside can suppress the growth and movement of lung cancer cells [28].

The GO enrichment analysis revealed that MGF was primarily associated with kinase phosphorylation, tyrosine phosphorylation, peptide tyrosine modification, enhancement of protein serine/threonine kinase activity, and protein kinase B signaling. The signaling pathways enriched by KEGG mainly targeted the PI3K/AKT/MTOR pathway, Ras pathway, MAPK pathway, and apoptosis-related pathways. This further demonstrates that the mechanism by which fermented drugs combat lung cancer primarily involves the inhibition of lung cancer cell proliferation and the induction of apoptosis in these cells. The molecular docking findings also showed stable interactions with MAPK, EGFR, and Caspase9, further confirming that MGF can trigger cancer cell apoptosis by targeting cell membrane receptor factors and inhibiting cell proliferation.

## Figures and Tables

**Table 1:** Tentatively identified major components of MGF.

NO	EM	Tentative Identification	Adducts	Formula	Mass Error (ppm)
TC1	553.3134 *m*/*z*	11α-O-2-Methylbutyryl-12β-O-2-benzoyltenacigeninB	[M + H]^+^	C_33_H_44_O_7_	−4.69
TC2	531.3321 *m*/*z*	11α-O-2-Methylbutyryl-12β-O-2-tigloyltenacigeninB	[M + H]^+^	C_31_H_44_O_7_	−3.01
TC3	529.3150 *m*/*z*	11α, 12β-Di-O-tigloyltenacigeninB	[M + H]^+^	C_31_H_44_O_7_	−2.45
TC4	551.2981 *m*/*z*	11α-O-Tigloyl-12β-O-benzoyl-marsdenin	[M + H]^+^	C_33_H_42_O_7_	−3.99
TC5	529.3147 *m*/*z*	11α,12β-O,O-Ditigloyl-17β-tenacigenin B	[M + H]^+^	C_31_H_44_O_7_	−2.45
TC6	511.2696 *m*/*z*	11α-O-Benzoyl-12β-O-acetyltenacigenin B	[M + H]^+^	C_30_H_38_O_7_	−3.71
TC7	489.2840 *m*/*z*	11α-O-Tigloyl-12β-O-acetyltenacigenin B	[M + H]^+^	C_28_H_40_O_7_	−1.43
TC8	447.2730 *m*/*z*	11α-O-Tigloyl-17β-tenacigenin B	[M + H]^+^	C_26_H_38_O_6_	−2.45
TC9	383.2418 *m*/*z*	Tenacigenin C	[M + H]^+^	C_26_H_38_O_7_	−2.34
TC10	363.2174 *m*/*z*	Tenacigenin A	[M − H]^−^	C_21_H_32_O_5_	−0.55
TC11	363.2166 *m*/*z*	Tenacigenin B	[M − H]^−^	C_21_H_32_O_6_	−2.75
TC12	837.4424 *m*/*z*	Tenacissoside I	[M + Na]^+^	C_44_H_62_O_14_	−0.95
TC13	815.4134 *m*/*z*	Tenacissoside G	[M + Na]^+^	C_42_H_64_O_14_	−1.34
TC14	829.4216 *m*/*z*	Tenacissimoside H	[M − H]^−^	C_47_H_76_O_22_	−0.12

## Data Availability

Data are contained within this article.

## Abbreviations

MGF	*Ganoderma lucidum* and *Marsdenia tenacissima*
TCGA	The Cancer Genome Atlas Program
LUAD	lung adenocarcinoma
AUC	Area Under the Curve
TCMSP	Traditional Chinese Medicine Systems Pharmacology
PPI	Protein–Protein Interaction
KEGG	Kyoto Encyclopedia of Genes and Genomes
GO	Gene Ontology
PI3K	Phosphatidylinositol 3-kinase
Rap	Ras-related protein
PCA	Principal Component Analysis
mTOR	mammalian target of rapamycin
Ras	rat sarcoma
Casp3	Caspase-3
Casp9	Caspase-9
EGFR	epidermal growth factor receptor
VEGF	vascular endothelial growth factor
MAPK1	mitogen-activated protein kinase 1
BP	biological process
CC	Cellular Component
MF	molecular function
ROC	Receiver Operating Characteristic
